# Antioxidant and Cytoprotective Activities of *Fucus spiralis* Seaweed on a Human Cell in Vitro Model

**DOI:** 10.3390/ijms18020292

**Published:** 2017-01-29

**Authors:** Susete Pinteus, Joana Silva, Celso Alves, André Horta, Olivier P. Thomas, Rui Pedrosa

**Affiliations:** 1 MARE—Marine and Environmental Sciences Centre, School of Tourism and Maritime Technology, Polytechnic Institute of Leiria, 2520-641 Peniche, Portugal; susete.pinteus@ipleiria.pt (S.P.); joana.m.silva@ipleiria.pt (J.S.); celso.alves@ipleiria.pt (C.A.); andre.horta@ipleiria.pt (A.H.); 2 Marine Biodiscovery, School of Chemistry, National University of Ireland Galway, University Road, H91TK33 Galway, Ireland; olivier.thomas@nuigalway.ie

**Keywords:** edible algae, oxidative stress, phlorotannins, marine natural products, MCF-7 cells, reactive oxygen species

## Abstract

Antioxidants play an important role as Reactive Oxygen Species (ROS) chelating agents and, therefore, the screening for potent antioxidants from natural sources as potential protective agents is of great relevance. The main aim of this study was to obtain antioxidant-enriched fractions from the common seaweed *Fucus spiralis* and evaluate their activity and efficiency in protecting human cells (MCF-7 cells) on an oxidative stress condition induced by H_2_O_2_. Five fractions, F1–F5, were obtained by reversed-phase vacuum liquid chromatography. F3, F4 and F5 revealed the highest phlorotannin content, also showing the strongest antioxidant effects. The cell death induced by H_2_O_2_ was reduced by all fractions following the potency order F4 > F2 > F3 > F5 > F1. Only fraction F4 completely inhibited the H_2_O_2_ effect. To understand the possible mechanisms of action of these fractions, the cellular production of H_2_O_2_, the mitochondrial membrane potential and the caspase 9 activity were studied. Fractions F3 and F4 presented the highest reduction on H_2_O_2_ cell production. All fractions decreased both caspase-9 activity and cell membrane depolarization (except F1). Taken all together, the edible *F. spiralis* reveal that they provide protection against oxidative stress induced by H_2_O_2_ on the human MCF-7 cellular model, probably acting as upstream blockers of apoptosis.

## 1. Introduction

For thousands of years, edible seaweeds have been highly valued and widely consumed as a direct human food by Oriental communities [1]. According to several studies, these organisms contain a range of components that have potential health benefits. They are also good sources of dietary fiber, polyunsaturated fatty acids, minerals, vitamins and a wide range of phenolic compounds [2,3]. Marine brown algae include a wide range of edible seaweeds such as *Laminaria* spp., *Undaria* spp., *Ecklonia* spp., *Sargassum* spp., *Fucus* spp., etc., which are rich in a specific group of antioxidant compounds, the phlorotannins [1,4,5]. Phlorotannins are unique phenolic compounds that belong to a large class of marine secondary metabolites exclusively produced by brown algae. They are often considered to act as a chemical defense against herbivores and also possess primary functions, such as contributing to cell wall structure and reproduction [6]. These potent antioxidant compounds are oligomers or polymers of phloroglucinol (1,3,5-trihydroxybenzene), connected by aryl–aryl bonds (fucols), ether bonds (phlorethols, hydroxyphlorethols, fuhalols), or both (fucophlorethols), or with a dibenzodioxin linkage (eckols and carmalols) [7].

Due to the health benefits of phlorotannins, marine brown algae are considered a rich source of healthy food. In fact, in Occidental cultures, algae are highly valued for their nutritional content as well as antioxidant benefits [8,9].

Antioxidants act as sacrificial reducing agents, eliciting their benefits by preventing, delaying, or neutralizing the effects of oxidative change and suppression and/or scavenging of free radicals. Therefore, antioxidants can neutralize reactive free radicals in cells, reducing potential mutations and act as stabilizers in the food industry, in order to increase the shelf-life of food products [10,11]. Consequently, antioxidant compounds have attracted the interest of many researchers, mainly for recognition of their scavenging properties, and also for their preventive role in several diseases associated with oxidative stress. Oxidative stress occurs when the balance between antioxidants and reactive oxygen species (ROS) is disrupted because of either depletion of antioxidants or accumulation of ROS. ROS are produced by cellular metabolic activities and triggered by environmental factors, such as air pollutants or cigarette smoke. They are highly reactive and can react with several biological macromolecules in cells, such as carbohydrates, nucleic acids, lipids and proteins, altering their functions [12]. Generation and accumulation of ROS are, therefore, detrimental to cells and they are proven to be directly related to the occurrence of a vast range of disorders/diseases from neurodegenerative diseases, cancer to skin problems, and are even linked to the aging process [13,14,15].

One approach for preventing or treating these ROS-mediated disorders can be based on a diet rich in antioxidants. Nevertheless, while many antioxidants have revealed high efficiency in vitro, their efficiency is unclear in humans [15]. Therefore, the search for new molecules with antioxidant properties from natural sources is of great importance.

*Fucus spiralis* (spiral wrack) is an edible brown seaweed (Phaeophyceae) living on the Atlantic coasts of Europe and North America and has been previously identified for high antioxidant properties, mainly linked to its phlorotannin content [16,17,18]. The separation of seaweed’s phenolic compounds is usually highly challenging due to their instability, complex mixture, pH dependence and high affinity with other biomolecules. In this work, we wanted to obtain phlorotannin-enriched fractions from *Fucus spiralis* and to assess their antioxidant activity and efficiency in protecting human cells (MCF-7 cells) exposed to oxidative stress conditions.

## 2. Results

### 2.1. Antioxidant Activity

In order to describe the antioxidant potential of each fraction, the total phenolic content (TPC) was assessed as well as the evaluation of the Oxygen Radical Absorbance Capacity (ORAC), the 2,2-diphenyl-1-picrylhydrazyl (DPPH) radical scavenging capacity and evaluation of the hydroxyl radical scavenging activity (^•^OH).

The total phenolic content was evaluated in all fractions by the Folin–Ciocalteu method, and the results are shown in Table 1. Fractions 4, 3 and 5 demonstrated the highest phenolic content with 419.00 ± 3.00, 379.00 ± 34.0 and 285.00 ± 12.00 mg·PE/g extract, respectively.

As regards the DPPH radical scavenging ability, all fractions presented a concentration-dependent effect and fraction 5 presented the highest scavenging activity with an IC_50_ of 9.74 µg/mL (8.14–11.66) followed by fractions 4 and 3, which presented an IC_50_ of 13.94 µg/mL (11.13–17.46) and 15.58 µg/mL (13.31–18.22), respectively.

Regarding the hydroxyl radical scavenging activity, all fractions presented a concentration-dependent effect, and fraction 1 presented the highest potential in this radical scavenging activity with an IC_50_ of 7.90 µg/mL (5.60–11.60) followed by fractions 3, 4 and 2, which presented an IC_50_ of 9.73 µg/mL (6.51–14.55), 10.86 µg/mL (5.90–19.95) and 11.52 µg/mL (8.03–16.52), respectively.

As regards ORAC, all fractions presented a high oxygen radical scavenging capacity, ranging from 2988.00 to 34,893.68, fraction 4 being the strongest scavenger, followed by fractions 5 and 3 with 30,691.00 and 6877.00 µmol·TE/g extract, respectively (Table 1).

**Figure 1:**
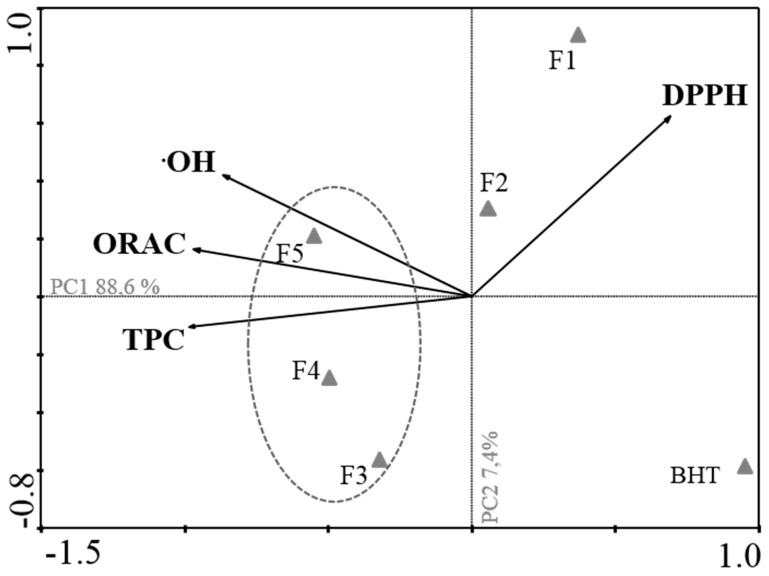
Principal component analysis (PCA) of total phenolic content (TPC) and antioxidant activities (DPPH, ^•^OH and ORAC) of *Fucus spiralis* fractions. The vectors represent a variant existing among the antioxidant methods. The first principal component (PC1) and the second principal component (PC2) represent the total variance of the data. The circle defines a group of samples that contains the highest antioxidant activity. The triangles in the figure represent each sample distributed according to its variance.

A principal components analysis (Figure 1) was performed in order to obtain an overview of the similarities and differences between all the studied fractions, as well as to investigate the relationship between the different methods used for evaluating the antioxidant activity.

The first two principal components, PC1 and PC2, explain 88.6% and 7.4% of the total variance of the data set, respectively. Through the analysis of PC1, it is possible to observe a strong negative correlation between TPC (on the left) and DPPH (on the right), indicating that fractions with the highest potency in scavenging DPPH radical (lower IC_50_ values) presented the highest TPC, namely, fractions 3, 4 and 5. On the other hand, TPC shows a positive correlation with ORAC, revealing that fractions with the highest Phloroglucinol-equivalent contents were also the ones presenting the highest ORAC activity (fractions 4, 5 and 3). All fractions presented a high ^•^OH scavenging activity, being strongly correlated with ORAC and TPC. DPPH radical scavenging activity is not correlated with ORAC and ^•^OH scavenging. This analysis confirmed that F3, F4 and F5 are the most potent antioxidant fractions.

### 2.2. Protective Effect of Seaweed Fractions on MCF-7 Cells Exposed to H_2_O_2_

The antioxidant screening allowed us to verify that all fractions presented potential as ROS scavengers. Therefore, it was important to access their cytotoxicity on MCF-7 cells. The cytotoxicity of all fractions was evaluated by the MTT (3-(4,5-dimethylthiazol-2-yl)-2,5-diphenyl tetrazolium bromide) method, and none of the fractions (1 mg/mL) presented cytotoxicity on MCF-7 cells.

**Figure 2:**
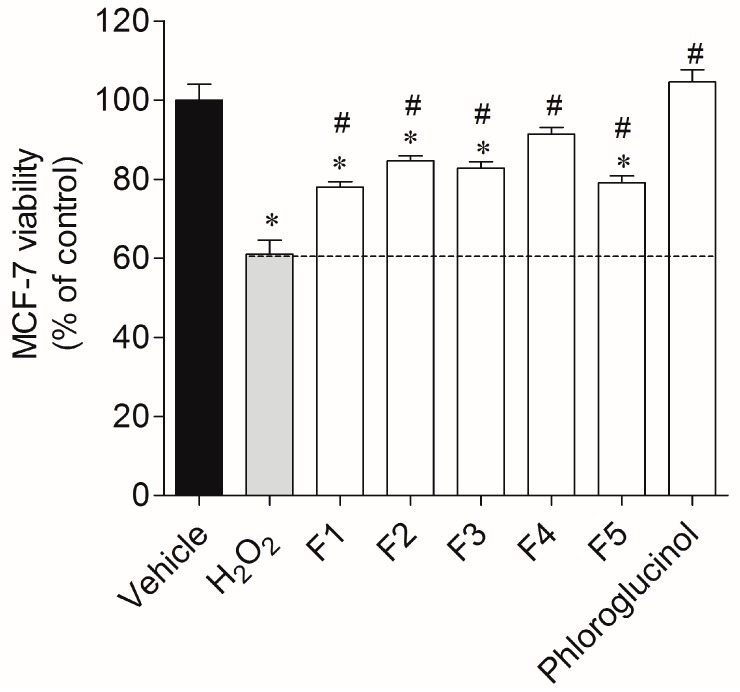
The effect of *Fucus spiralis* fractions (1 mg/mL) in an oxidative stress condition promoted by H_2_O_2_ (0.2 mM), after 24 h of treatment, on MCF-7 cells. Results were obtained by the MTT method. Values in each column represent the mean ± standard error of the mean (SEM) of eight independent experiments. Symbols represent statistically significant differences (*p* < 0.05, analysis of variance (ANOVA), Dunett’s test) when compared to: * vehicle and # to H_2_O_2_.

In the cytoprotection assay, MCF-7 cells were exposed to H_2_O_2_ (0.2 mM), which led to a 40% reduction of cell viability (61.1% ± 3.5%, Figure 2). On the other hand, in the presence of all fractions it was possible to verify a recovery of cell viability from 17%–30% after 24 h of treatment, with fraction 4 presenting the highest protective effect with 91.4% ± 1.7% of viable cells (30% recovery). Phloroglucinol was used as standard and fully blunted the H_2_O_2_ effect (100% ± 3.13% viable cells).

### 2.3. Study of the Cellular Mechanisms Involved in the Cytotoxicity Induced by H_2_O_2_ on MCF-7 Cells in the Presence or Absence of Fucus spiralis Fractions

#### 2.3.1. Real-Time Quantification of H_2_O_2_ Production

**Figure 3:**
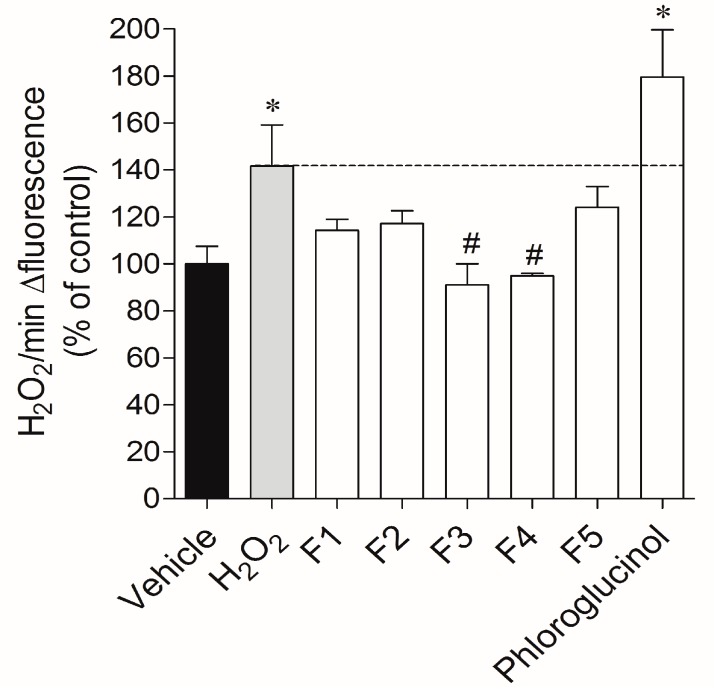
Real-time H_2_O_2_ quantification on MCF-7 cells in the absence or presence of *Fucus spiralis* fractions (1 mg/mL; 24 h). Each column represents the mean ± standard error of the mean (SEM) of three or four independent experiments. Symbols represent statistically significant differences (*p* < 0.05, ANOVA, Dunett’s test) when compared to: * vehicle and # to H_2_O_2_.

In order to understand whether the cytotoxicity induced by H_2_O_2_ and the protection evidenced by *Fucus spiralis* fractions were associated with oxidative stress, H_2_O_2_ production was quantified in real time. The exposure of MCF-7 cells to H_2_O_2_ (0.2 mM) during 24 h promoted an increase of H_2_O_2_ levels in about 42% when compared with the vehicle (Figure 3). This increase was also visible after the replacement of the H_2_O_2_ loading medium. On the other hand, when cells were incubated with H_2_O_2_ in the presence of fractions 3 and 4, the H_2_O_2_ production decreased by 50.64% (91.04% ± 8.96% of control) and 46.71% (94.97% ± 1.08% of control), respectively. Fractions 1, 2, 5 and phloroglucinol did not exhibit capacity to decrease the H_2_O_2_ production.

#### 2.3.2. Mitochondrial Membrane Potential (ΔΨm)

**Figure 4:**
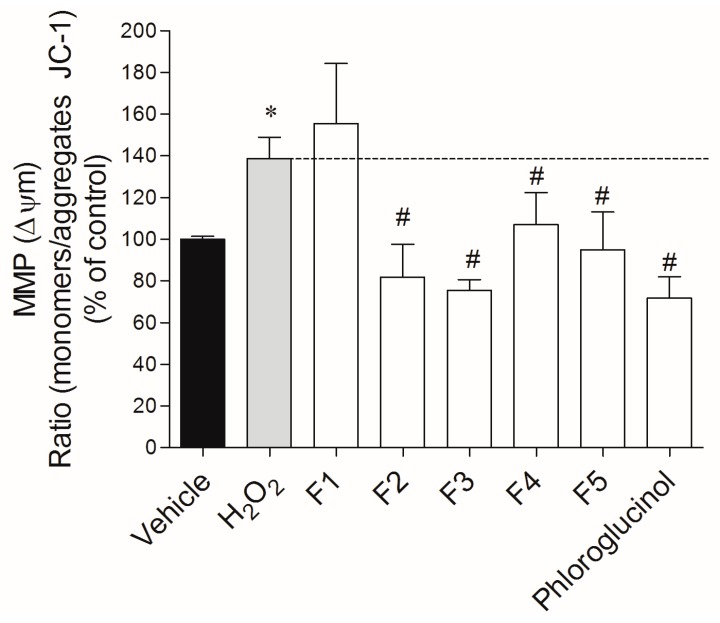
H_2_O_2_ (0.2 mM) effects on the mitochondrial membrane potential (MMP) of MCF-7 cells in the absence or presence of *Fucus spiralis* fractions (1 mg/mL) after 24 h of treatment. The results were obtained by the ratio between the monomers/aggregates of JC-1. The values in each column represent the mean ± standard error of the mean (SEM) of seven or eight independent experiments. Symbols represent statistically significant differences (*p* < 0.05, ANOVA, Dunett’s test) when compared to: * vehicle and # to H_2_O_2_.

The incubation of MCF-7 cells with H_2_O_2_ (0.2 mM) induced a depolarization of the mitochondrial membrane potential (ΔΨm, MMP) in about 40% compared with vehicle, after 24 h of incubation (Figure 4). During the treatment, it was possible to observe a preventive effect of all fractions, with the exception of fraction 1. The H_2_O_2_-induced depolarization of MMP was reduced in 63.3%, 56.9%, 43.8% and 31.6% by fractions 3, 2, 5 and 4, respectively. Phloroglucinol was used as standard and presented a protective effect of about 67%.

#### 2.3.3. Caspase-9 Activity

**Figure 5:**
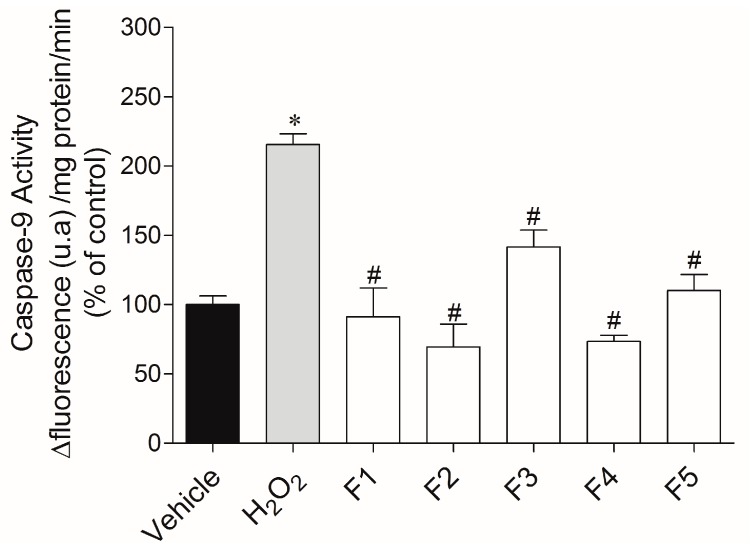
H_2_O_2_ (0.2 mM) effects on caspase-9 activity of MCF-7 cells in the presence or absence of *Fucus spiralis* fractions (1 mg/mL) after 24 h of treatment. The activity was quantified by the slope of the linear phase accumulation of 7-amino-4-(trifluoromethyl) coumarin (between 25 and 75 min). The results are presented in arbitrary units of fluorescence per mg of protein (% of control). The values in each column represent the mean ± standard error of the mean (SEM) from three independent experiments. Symbols represent statistically significant differences (*p* < 0.05, ANOVA, Dunett’s test) when compared to: * vehicle and # to H_2_O_2_.

In order to understand whether the cell death induced by H_2_O_2_ was linked to apoptosis, the caspase-9 activity was evaluated (Figure 5). When MCF-7 cells were exposed to H_2_O_2_, we observed an increase of caspase-9 activity in more than two times (215.50% ± 7.78%) when compared to the vehicle (100.00% ± 6.24%). When cells were incubated with H_2_O_2_ and fractions (1 mg/mL; 24 h), it was possible to detect a reduction of caspase-9 activity for all fractions when compared to the H_2_O_2_ situation.

## 3. Discussion

Oxidative stress has been associated with the occurrence of numerous diseases [14,19]. Although different defense mechanisms against ROS exist, the aging process associated with an unhealthy lifestyle can trigger ROS production and induce their effects. Therefore, preventing ROS formation as well as fortifying the natural defense mechanisms can be beneficial in the prevention and/or treatment of several metabolic disorders [20].

During the last decade, marine organisms have been targeted for their ability to produce bioactive compounds with high antioxidant potential. Within marine organisms, algae are known to produce important bioactive compounds such as antimicrobial, antitumor and antioxidant compounds. Nevertheless, the purification of bioactive compounds, especially antioxidant compounds of a phenolic nature, continues to be a challenging task. In this work, we fractionated the chemical extract of the seaweed *Fucus spiralis*, which was previously identified as a producer of compounds with high antioxidant activity [16,21].

In order to access the antioxidant activity of the fractions, different approaches were undertaken, namely DPPH radical scavenging activity, the oxygen radical absorbent capacity (ORAC), the hydroxyl radical scavenging activity and the quantification of the total phenolic content (TPC) was also accessed. All fractions presented high antioxidant activity; however, it was important to compare all the data to identify the most potent fractions. Through principal component analysis (PCA), it was possible to verify that fractions F4, F5 and F3 are the most bioactive fractions as regards the antioxidant activity, and therefore the most promising fractions for protecting from ROS effects. Nevertheless, since all of the fractions presented high antioxidant activity, they were all tested for cytotoxicity and for their ability to revert an oxidative stress condition induced by H_2_O_2_ on a cellular model, the MCF-7 cells.

Although H_2_O_2_ is a relatively weak oxidant compared to other ROS such as ^•^OH, it has emerged as an important signaling molecule based on its unique biochemical properties. It is generated from nearly all sources of oxidative stress. Exogenous H_2_O_2_ can also enter cells readily due to its high membrane permeability, inducing cytotoxicity. It is ubiquitously present in the biological system with a relatively long half-life and it is soluble in both lipid and aqueous media [22,23]. Moreover, H_2_O_2_ can form highly reactive and detrimental ^•^OH species through the metal-catalyzed Fenton reaction, which could involve numerous targets, ultimately causing oxidative injury in cells [24]. Therefore, H_2_O_2_ was used to induce oxidative stress in the model with MCF-7 cells in the present study.

Our results clearly demonstrated that all fractions presented capacity to revert a stress situation promoted by the H_2_O_2_. On the other hand, through the analysis of the real-time H_2_O_2_ production, only fractions 3 and 4 were able to decrease the production of H_2_O_2_ when compared to the control. Despite its toxicity, H_2_O_2_ is normally produced by healthy cells in response to activation of various cell surface receptors and it plays a key role as an intracellular messenger in mammalian cells. For instance, cells are known to produce H_2_O_2_ to signal the immune system to heal wounds and mediate cell-to-cell communication [23]. Nevertheless, cancer cells keep producing H_2_O_2_ and do not regulate its production. Our results highlight the potential of *Fucus spiralis* phlorotannin-enriched fractions as ROS protectors, by increasing cell viability in the presence of the H_2_O_2_ radical and, on the other hand, by decreasing the cellular production of H_2_O_2_. Interestingly, phloroglucinol did not reduce H_2_O_2_ production, suggesting that this antioxidant molecule can be involved in the detoxification of other reactive molecules resulting from H_2_O_2_ transformation. Other investigations support the potential of phlorotannins as ROS protectors in H_2_O_2_-induced oxidative stress. Kang and co-workers [25] evaluated the cytoprotective effect of eckol, a phlorotannin isolated from *Ecklonia cava* (a brown seaweed) on an oxidative stress condition induced by H_2_O_2_ on lung fibroblast cells (V79-4) and they could verify an effective cellular protection by decreasing ROS levels. On the other hand, a recent study on *Ecklonia cava* also reported the protective effects of phlorotannins against H_2_O_2_-induced oxidative stress in murine hippocampal HT22 cells [26]. Heo and Jeon [27] reported the protective effect of a phlorotannin (diphlorethohydroxycarmalol) isolated from *Ishige okamura* on a monkey kidney fibroblast cells line (Vero). The results clearly indicated that diphlorethohydroxycarmalol exerted profound protective effects against H_2_O_2_-mediated cell damage. These works corroborate our data showing clear evidence of phlorotannins cytoprotective potential in oxidative stress situations. Furthermore, several pathologies are associated with oxidative stress conditions, such as cancer. This idea put in incidence the *F. spiralis* protection against oxidative stress, mainly through the phlorotannin-enriched fractions. This view is supported by the study of Kim et al. (2015) [28], which demonstrated that the phlorotannin dieckol derived from *Ecklonia cava* inhibited the MCF-7 cell migration through inhibition of matrix metalloprotease (MMP)-9 and vascular endothelial growth factor (VEGF).

In order to understand the mechanisms that can be involved in cellular protection, the mitochondrial membrane potential (ΔΨm) and caspase-9 activity were examined. Mitochondria plays a key role in the apoptotic process due to being involved in two distinct signaling pathways: (1) maintenance of ATP production, and (2) mitochondrial membrane potential and mitochondrial membrane permeability. The mitochondrial membrane potential results from the difference in the electrical potential generated by the electrochemical gradient across the inner membrane, which is critical for maintaining the physiological function of the respiratory chain to generate ATP. Therefore, changes in the ΔΨm have been originally postulated to be early and obligate events in the apoptotic signaling pathway [29,30]. The H_2_O_2_ treatments promoted a membrane depolarization, which, consequently, may be involved in the apoptotic events, which led to the cell death verified. In the presence of *Fucus spiralis* fractions, the depolarization induced by H_2_O_2_ was completely prevented by all fractions, except F1. Taking into account that apoptosis-signaling cascades can be initiated by mitochondria, the maintenance of the membrane potential and mitochondrial functionality in H_2_O_2_-exposed cells by the tested fractions can explain the increased cells survival. Supporting our hypothesis of phlorotannins as mitochondrial membrane protectors, Zhang and co-workers [31] also verified that a phlorotannin isolated from *Ecklonia cava* (triphlorethol-A) prevented cellular damage by restoring the mitochondrial membrane potential of hamster lung fibroblast (V79-4) cells, which were previously distressed with formaldehyde.

The apoptotic process is triggered by a sequential activation of caspases. Caspase-9 is activated in the upstream of the apoptotic process by the release of cytochrome c by the mitochondria as a response to an apoptotic stimulus. Once activated, caspase-9 then cleaves and activates other downstream caspases such as caspase-3 [32].Our results show that all fractions decreased caspase-9 activity. In accordance with this, Chia and co-workers [33] analyzed the antioxidant and cytotoxic activities of three species of tropical seaweed extracts, and after 24 h of incubation, *Turbinaria ornata*, a brown macroalgae of the order Fucales, was able to decrease the caspase-9 activity also on MCF-7 cells. Another work with phlorotannins (2-phloroeckol and eckol) from *Ecklonia cava* revealed their protective effects on oxidative stress induced by Tacrine on HepG2 cells, by inhibiting the activation of caspase-3 [34]. Taken altogether, our results suggest that the cell death promoted by H_2_O_2_ is mainly mediated by apoptosis, and that *Fucus spiralis* fractions have the capacity to inhibit cellular damage promoted by ROS. These protective effects can be very useful in the prevention and treatment of free radical-related diseases.

## 4. Materials and Methods

### 4.1. Chemicals and Reagents

Methanol, *n*-hexane, water and dichloromethane of analytical grade were purchased from Fisher Scientific (Loughborough, Leicestershire, UK). All of the other chemicals and reagents were purchased from Sigma (Sigma-Aldrich GmbH, Steinheim, Germany).

### 4.2. Collection, Preparation and Extraction of Fucus spiralis

*Fucus spiralis* specimens were collected between April and July of 2014, in Marques Neves beach (39°37′03.53″ N 9°38′87.59″ W), Peniche (Portugal) and immediately transferred to laboratory. The samples were then soaked in seawater to remove epibionts, and finally freeze-dried (Scanvac Cool Safe, LaboGene, Lynge, Denmark). Dry samples were extracted overnight with constant stirring in a 1:40 biomass:solvent ratio with methanol. The organic extract was subjected to a liquid-liquid partition with *n*-hexane to remove fats. The methanol fraction was then concentrated in a rotary evaporator at 40 °C and the biomass was stored at −20 °C until further use.

### 4.3. Fractionation of Fucus spiralis Methanolic Extract by Vacuum Liquid Chromatography (VLC)

In order to concentrate the antioxidant metabolites, a reversed-phase VLC was performed with the following solvent system (400 mL each): 100% H_2_O (F1); 1:1 H_2_O:CH_3_OH (F2); 100% CH_3_OH (F3); 3:1 CH_3_OH:CH_2_Cl_2_ (F4) and 100% CH_2_Cl_2_ (F5) on a pad of C_18_ bulk (Polygoprep 60–50 Macherey-Nagel, GmbH, Düren, Germany). The dried fractions were then stored at −20 °C and used in the experimental assays.

### 4.4. Analysis of Total Phenolic Content (TPC)

The total phenolic content of *Fucus spiralis* fractions was determined using the Folin–Ciocalteu method adapted to microscale [35] with minor modifications. Phloroglucinol was used as standard. Briefly, 2 µL of extract was added to 158 µL of distilled water in a 96-well microplate, followed by 10 µL of Folin-Ciocalteu reagent. The reaction mixture was pre-incubated for 2 min at room temperature and then 30 µL of 20% Na_2_CO_3_ (*w*/*v*) was added and mixed. After one hour of reaction in the dark, the absorbance was measured at 755 nm (Synergy H1 Multi-Mode Microplate Reader, BioTek^®^ Instruments, Winooski, VT, USA) against blank solution (prepared by the same procedure described above, but replacing Folin-Ciocalteu reagent for the same amount of water) and used to calculate the phenolic content. The TPC is expressed as milligrams of phloroglucinol equivalents per gram of dry extract (mg·PE/g).

### 4.5. Evaluation of Antioxidant Activities

#### 4.5.1. DPPH (1,1-Diphenyl-2-picrylhydrazyl) Radical Scavenging Activity

DPPH radical scavenging activity was performed according to Brand–Williams and co-workers [36] and adapted to microscale with slight modifications. DPPH radical was dissolved in absolute ethanol. Various concentrations (10–1000 μg/mL) of 2 µL of sample solution were added to 198 µL of the DPPH radical solution (0.1 mM). The mixture was vortexed for 1 min and allowed to stand at room temperature in the dark for 30 min, at which time the decrease in absorbance at 517 nm was measured. The radical solution was freshly prepared each day. BHT (butylated hydroxytoluene) was used as a standard. The ability of samples to scavenge the DPPH radical was calculated using the follow equation:

(1) $$\mathrm{DPPH}\;\mathrm{radical}\;\mathrm{scavenging}\;\mathrm{activity}\;(\%\;\mathrm{of}\;\mathrm{control})\;=\;\left[1\;-\;\left(\frac{A_{\mathrm{sample}}\;-\;A_{\mathrm{sample}\;\mathrm{blank}}}{A_{\mathrm{control}}}\right)\right]\;\times \;100$$

where the A_control_ is the absorbance of the control (DPPH solution with dimethyl sulfoxide), the A_sample_ is the absorbance of the test sample (DPPH solution plus test sample), and the A_sample blank_ is the absorbance of the sample in ethanol (sample without DPPH solution). Results are expressed as mean ± SEM (standard error of the mean). IC_50_ values (μg/mL) were also determined for the fractions that scavenged DPPH radical over 50%.

#### 4.5.2. Oxygen Radical Absorbance Capacity (ORAC)

Oxygen Radical Absorbance Capacity (ORAC-fluorescein) assay was performed as described by Dávalos and co-workers (2004). This method is based on the decrease of the fluorescence of a fluorescent probe (β-phycoerythrin) by reacting with peroxyl radicals generated by the decomposition of 2,2’-azobis(2-methylpropionamidine) dihydrochloride (AAPH). In the presence of antioxidants, the peroxyl radicals are scavenged and the decay on the fluorescence is delayed. The difference between the area under the fluorescence decay curve (AUC) corresponding to the mixture with antioxidant, and the AUC corresponding to the mixture without antioxidant, is then converted into a Trolox standard calibration. The antioxidant activity is expressed in µmol of Trolox equivalents/g of dry extract (µmol·TE/g).

The reaction was carried out in 75 mM phosphate buffer (pH 7.4) on a total volume of 200 µL. Briefly to 20 µL of sample was added 120 µL of fluorescein (70 nM in phosphate buffer). After being pre-incubated for 15 min at 37 °C, an AAPH solution (60 µL; 12 mM, final concentration) was added. The fluorescence (λ_excitation_ = 458 nm, λ_emission_ = 520 nm) was recorded every minute for 240 min. A blank using phosphate buffer instead of the fluorescein, and eight calibration solutions using Trolox (1–8 µM), were also carried out in each assay. All reactions were prepared in duplicate, and at least three independent assays were performed for each sample.

#### 4.5.3. Hydroxyl Radical Scavenging Activity (^•^OH)

Hydroxyl radical (^•^OH) scavenging activity was measured by the capacity of the seaweed fractions to scavenge the ^•^OH generated by the Fe^3+^–ascorbate–EDTA–H_2_O_2_ system (Fenton reaction) [37,38]. The ability to eliminate hydroxyl radicals was measured by the method adapted from Halliwell and co-workers (1987) and Kunchandy and Rao (1990) [39]. The reaction mixture consisted in 200 µL EDTA (1.04 mM), 200 µL FeCl_3_ (0.2 mM) (1:1 *v*/*v*), 100 µL of H_2_O_2_ (1.0 mM), 100 µL of ascorbic acid (1.0 mM), 100 µL of 2-deoxy-d-ribose (28 mM in 20 mM KH_2_PO_4_-KOH buffer, pH 7.4) and 500 µL of the sample. After 1 h incubation at 37 °C, 1.0 mL of thiobarbituric acid (1%) and 1.0 mL of trichloroacetic acid (2.8%) are added and the solution incubated at 100 °C for 20 min. After cooling, absorbance is measured at 532 nm, against a blank sample.

The ability of test samples to scavenge the ^•^OH was calculated using the follow equation:

(2) $$\mathrm{OH}\;\mathrm{scavenging}\;\mathrm{activity}\;(\%\;\mathrm{of}\;\mathrm{control})\;=\;\left[1\;-\;\left(\frac{A_{\mathrm{sample}}\;-\;A_{\mathrm{sample}\;\mathrm{blank}}}{A_{\mathrm{control}}}\right)\right]\;\times \;100$$

where the A_control_ is the absorbance of the control (without sample), the A_sample_ is the absorbance in the presence of the sample, and the A_sample blank_ is the absorbance of the solution without 2-deoxy-d-ribose. Results are expressed as mean ± SEM. IC_50_ values (μg/mL) were also determined for the fractions that scavenged ^•^OH over 50%.

### 4.6. *In Vitro* Assay of Oxidative Stress Prevention

#### 4.6.1. Cell Maintenance Culture Conditions

Human breast adenocarcinoma model (MCF-7 cells-ACC 115) was acquired from the DSMZ—German collection of microorganisms and cell cultures. The cells were cultured in RPMI 1640 medium supplemented with 10% of fetal bovine serum (FBS), 1% of antibiotic and antimicotic solution (100 U/mL penicillin G, 0.25 µg/mL amphotericin B and 100 µg/mL streptomycin), 1% of MEM non-essential amino acids, 1 mM of sodium pyruvate, 10 µg/mL of human insulin. Cells medium was changed every three days, and the cells reached confluence after 5–6 days of initial seeding. For subculture, cells were dissociated with trypsin-EDTA, split 1:3 and subculture in Petri dishes with 25 cm^2^ growth area. Cells were maintained at 37 °C, 95% of humidity and 5% of CO_2_ (CO_2_ Unitherm, Planegg, Munich, Germany).

#### 4.6.2. *Fucus spiralis* Fractions Cytotoxicity Evaluation

The cytotoxicity of seaweed fractions with major antioxidant activity was determined. Cell viability studies were performed after cells reached the total confluence in 96-well plates (Thermo Fisher Scientific, Seoul, Korea). The selected fractions were dissolved in culture medium without FBS (1 mg/mL) and sterile filtered (0.2 µm, Whatman, Buckinghamshire, UK). Cells were incubated during 24 h and cell viability evaluated by the MTT (3-(4,5-dimethylthiazol-2-yl)-2,5-diphenyl tetrazolium bromide) method [40].

#### 4.6.3. Evaluation of the Protective Effect of Seaweed Fractions in an Oxidative Stress Condition Induced by H_2_O_2_ on MCF-7 Cells

Seaweed fractions with high antioxidant activity and without cytotoxicity were evaluated for their potential in preventing an oxidative stress condition promoted on MCF-7 cells by the addition of H_2_O_2_ (0.2 mM). The concentration of the extracts and H_2_O_2_ and the assay conditions were defined in preliminary tests, and established as follows: after cells reached total confluence in 96-well plates, previously filtered (0.2 µm) seaweed fractions (1 mg/mL on culture medium without FBS) were added together with H_2_O_2_ (0.2 mM). The incubation occurred along 24 h. The protective effects of seaweed fractions were considered as MCF-7 cells viability comparing with the control (cells incubated with H_2_O_2_ (0.2 mM) during 24 h without seaweed fractions).

#### 4.6.4. Real-Time Quantification of H_2_O_2_ Production

Quantification of H_2_O_2_ was performed using the “Amplex^TM^ Red hydrogen peroxide Assay” Kit A22188 (ThermoFisher Scientific, Waltham, MA, USA). The amplex red is a fluorophore that evidences a low basal fluorescence, which reacts with H_2_O_2_ in a 1:1 ratio. This reaction is initiated with horseradish peroxidase, leading to the appearance of a highly fluorescent product, designated resofurin [41]. H_2_O_2_ production was quantified on MCF-7 cells after 24 h of treatment with H_2_O_2_ (0.2 mM) in the absence or presence of seaweed fractions (1 mg/mL). The variation of H_2_O_2_ production was accompanied in real-time along 60 min at room temperature. The fluorescence intensity was measured at wavelengths of 590 nm (excitation) and 530 nm (emission). The levels of H_2_O_2_ were calculated by the slope of the linear phase of fluorescence curve and the results were expressed in percentage of control.

#### 4.6.5. Mitochondrial Membrane Potential (ΔΨm)

Mitochondrial membrane potential was determined using the fluorescent probe, JC-1 (Molecular Probes, Eugene, OR, USA). MCF-7 cells were treated with H_2_O_2_ (0.2 mM) in the absence or presence of seaweed fractions (1 mg/mL) throughout 24 h. The medium was then removed, the cells washed with Hank’s buffer (medium composition in mM: NaCl 137, KCl 5, MgSO_4_ 0.8, Na_2_HPO_4_ 0.33, KH_2_PO_4_ 0.44, CaCl_2_ 0.25; MgCl_2_ 1.0 and Tris HCl 0.15, pH = 7.4), and incubated with JC-1 (3 µM) during 15 min at 37 °C. The JC-1 probe was removed and cells washed with Hank’s buffer. The formation of JC-1 aggregates (490 nm of excitation and 590 nm of emission) and the monomeric form of JC-1 (490 nm of excitation and 530 nm of emission) were measured simultaneously throughout 30 min. Results were expressed as the ratio of the monomers/aggregates of JC-1 in percentage of control.

#### 4.6.6. Caspase-9 Activity

Caspase-9 activity was assessed using “Caspase-9 Assay kit” K118 (Biovision, Milpitas, CA, USA). Cells were cultured in 6-well plates and treated with H_2_O_2_ (0.2 mM) in the presence or absence of *Fucus spiralis* VLC fractions (1 mg/mL) during 24 h. The cells were then washed twice with Hank’s buffer and collected by centrifugation at 5000 rpm during 10 min at 4 °C. The pellets were resuspended in 50 µL of lysis buffer and incubated on ice during 20 min. In order to separate the content of intracellular cytoplasmic organelles and cell membrane, centrifugation took place at 13,000 rpm during 20 min at 4 °C. Thereafter, 50 μL of supernatant was placed into a 96-well plate, to which was added 50 μL of reaction buffer containing DTT (10 mM) and 5 μL of substrate. This reaction was followed at wavelengths of 400 nm (excitation) and 505 nm (emission) along 90 min at room temperature. Caspase-9 activity was calculated by the slope of the fluorescence resulting from 7-amino-4-(trifluoromethyl) coumarin accumulation and expressed in % of control (Δ fluorescence (u.a)/mg of protein/min).

### 4.7. Statistical Analysis

Results were expressed as mean ± SEM. The IC_50_ concentration was calculated from nonlinear regression analysis using the GraphPad Prism software with the equation: *Y* = 100/(1 + 10^(*X* – LogIC50)^). All data were checked for normality and homoscedasticity using the Shapiro–Wilk and Levene’s test, respectively. Therefore, comparisons concerning variables, which did not meet variance or distributional assumptions, were carried out with Kruskal–Wallis non-parametric tests [42].

A two-step analysis was performed. The first step was a principal component analysis (PCA) for all antioxidant assays (TPC, DPPH, ORAC and ^•^OH), using CANOCO version 4.5 (Biometris–Plant Research International, Wageningen, The Netherlands) [43]. The PCA allowed for the detection of similarities between samples and for identifying the main associations among variables that are responsible for the total variability of the studied data. The PCA model was built on the average of the measured data, and full cross-validation was used to validate the model. All the components were analyzed. Also, one-way analysis of variance (ANOVA) was carried out in order to appraise the seaweed fractions effects (1 mg/mL) in an oxidative stress condition. The statistical comparisons among the groups were assessed through Newman–Keuls multiple comparison test [42]. Where applicable, results are presented as mean ± standard error of the mean (SEM). Differences were considered statistically significant at a level of 0.05 (that is, *p* < 0.05).

All calculations were performed with GraphPad InStat v. 3.5 (GraphPad Software, La Jolla, CA, USA).

## 5. Conclusions

Mitochondrial dysfunction and oxidative stress have been implicated in the pathophysiology of many diseases and, therefore, it is of great importance to promote the organism defenses by respecting an active lifestyle with a healthy diet. In this work, it was shown that the edible *Fucus spiralis* produces compounds that act on oxidative stress mechanisms, for instance, by reducing the H_2_O_2_ negative effects on cells, by maintaining the normal membrane potential and, also, by decreasing the caspase-9 activity which is involved in cell death mechanisms.

On the basis of these results, *Fucus spiralis* reveals to be an excellent source of natural antioxidant compounds with protective effects against oxidative stress.

## Figures and Tables

**Table 1:** Total phenolic content and antioxidant activity of *Fucus spiralis* fractions.

Fractions	TPC ^a^	DPPH ^b^	^•^OH ^c^	ORAC ^d^
1	8.00 ± 1.10	182.90 (124.30–269.00)	7.90 (5.60–11.60)	2,988.00 ± 107.77
2	33.00 ± 18.00	44.40 (38.30–51.47)	11.52 (8.03–16.52)	5,411.00 ± 91.54
3	379.00 ± 34.0	15.58 (13.31–18.22)	9.73 (6.51–14.55)	6,877.00 ± 92.56
4	419.00 ± 3.00	13.94 (11.13–17.46)	10.86 (5.90–19.95)	34,893.68 ± 945.20
5	285.00 ± 12.00	9.74 (8.14–11.66)	58.61 (41.32–83.14)	30,691.00 ± 1172.90
BHT	-	40.55 (25.74–63.87)	>1000	320.49 ± 32.31

^a^ mg Phloroglucinol equivalents/g extract; ^b^ 2,2-diphenyl-1-picrylhydrazyl (DPPH) radical scavenging activity (IC_50_ µg/mL); ^c^ hydroxyl (^•^OH) scavenging activity (IC_50_ µg/mL); ^d^ µmol TE/g extract; TPC = total phenolic content; ORAC = oxygen radical absorbance capacity; BHT = butylated hydroxytoluene.
